# Design, Synthesis, and Biochemical Evaluation of New Triazole Derivatives as Aurora-A Kinase Inhibitors

**DOI:** 10.3390/molecules26185678

**Published:** 2021-09-18

**Authors:** Omeima Abdullah

**Affiliations:** College of Pharmacy, Umm Al-Qura University, Makkah 21955, Saudi Arabia; oaabdullah@uqu.edu.sa

**Keywords:** cancer, Aurora-A, kinase, triazole

## Abstract

Aurora-A kinase, a key mitosis regulator, is expressed in a cell cycle-dependent manner and has an essential role in maintaining chromosomal stability and the normal progression of the cell through mitosis. Aurora-A kinase is overexpressed in many malignant solid tumors, such as breast, ovarian, colon, and pancreatic cancers. Thus, inhibiting Aurora-A kinase activity is a promising approach for cancer treatment. Here, new triazole derivatives were designed as bioisosteric analogues of the known inhibitor JNJ-7706621. The new compounds showed interesting inhibitory activity against Aurora-A kinase, as attested by IC_50_s in the low to submicromolar range.

## 1. Introduction

**Figure 1:**
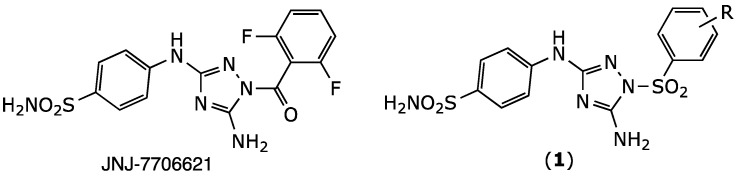
Chemical structure of JNJ-7706621 and its designed bioisosteric analogues (**1**).

Most types of cancers are characterized by genomic instability, which can range from subtle DNA sequence changes to gene amplification, chromosome translocations, and alterations in chromosome numbers [1,2]. Chromosome changes are generally referred to by the general term chromosomal instability. Chromosome stability depends on Aurora-A kinase, a member of the serine/threonine kinase family and a key mitosis regulator. Aurora-A kinase is expressed in a cell cycle-dependent manner and has an essential role in maintaining chromosomal stability and the normal progression of the cell through mitosis [3]. Aurora-A kinase is overexpressed in many malignant solid tumors, such as breast, ovarian, colon, and pancreatic cancers [4]. For this reason, inhibiting Aurora-A kinase activity is a promising approach for cancer treatment [5]. One compound, JNJ-7706621 (Figure 1), is an inhibitor of aurora kinases and of cyclin-dependent kinases [6]. JNJ-7706621 has shown potent antiproliferative activity in various cancerous cell lines and was several folds less potent at inhibiting normal cell growth [7]. Furthermore, it significantly reduced the tumor size in an A375 melanoma human tumor xenograft model [7]. Recently, NJ-7706621 promoted the reversal of resistance to CD37-targeted radioimmunotherapy in DLBC lymphoma cell lines [8]. In the present study, we report the synthesis of JNJ-7706621 analogues (**1**). These derivatives were designed using an isosteric approach where the amide bond of JNJ-7706621 is replaced by a sulfonamide function (Figure 1). The sulfonamide group was chosen in this study as it is considered highly druggable thanks to its improved stability to hydrolysis and hydrogen bonding potential [9].

## 2. Results and Discussion

### 2.1. Chemistry

**Figure 2:**
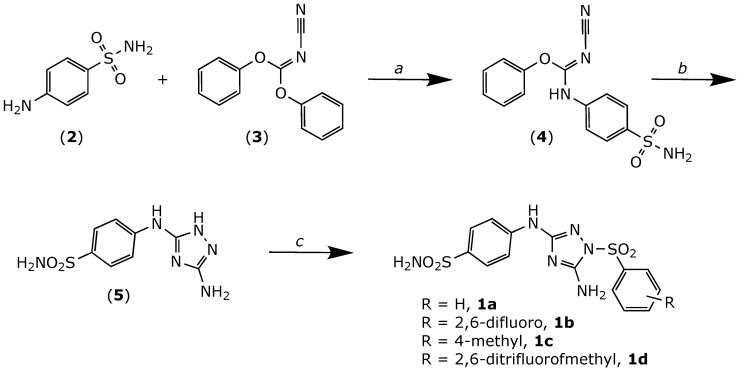
Chemical synthesis of compounds **1a–d**. Reagents and conditions: (a) iPrOH, heated in a sealed tube at 90 °C, 3 h. (b) NH_2_NH_2_, THF, RT, 12 h. (c) Pyridine, substituted sulfonyl chlorides, RT, overnight.

The compounds (**1a–d**) were obtained as shown in Figure 2. Briefly, the known triazole derivative (**5**) was synthesized as previously described [10], with slight modifications. First, 4-aminobenzensulfonamide (**2**) was heated in a sealed tube with diphenyl cyanocarbonimidate (**3**) at 90 °C to give intermediate (**4**). The latter was then cyclized by treatment with hydrazine to yield derivative 1H [1,2,4] triazole-3,5-diamine (**5**). Finally, the desired analogues were obtained by nucleophilic substitution of the corresponding phenylsulfonyl chlorides by the triazole derivative (**5**) using pyridine as a solvent.

### 2.2. Biological Evaluation

The inhibition of Aurora-A kinase activity by the designed compounds (Table 1) was evaluated by a method that involved the chelation-enhanced fluorescence mechanism (ChEF-based assay, PhosphoSens^®^) [11]. Compounds **1a–c** inhibited the kinase activity of Aurora-A kinase with submicromolar IC_50_s. However, the derivatives **1a–c** were tenfold less active compared to the reference compound JNJ-7706621. The electron-donating nature of the phenyl substituents does not seem to have an important effect on the inhibitory activity, since no significant difference was noted between non-substituted (**1a**), difluoro (**1b**), and methyl derivatives (**1c**). Nevertheless, the bulky trifluoromethyl group seems to cause unfavorable steric interactions.

### 2.3. Molecular Modeling

**Figure 3:**
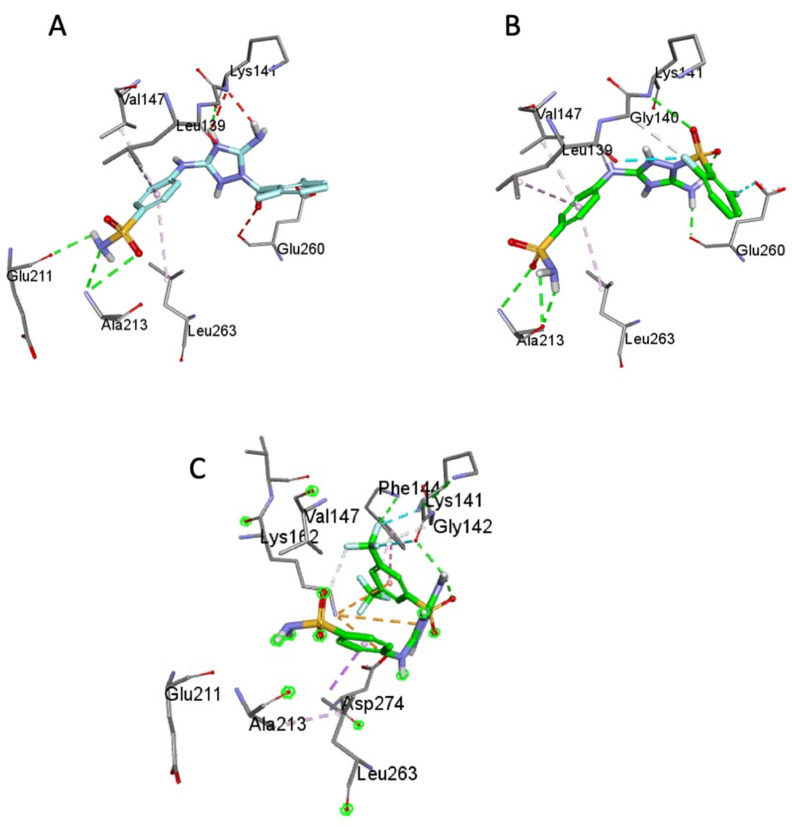
The molecular interactions of JNJ-7706621 (**A**), **1b** (**B**) and **1d** (**C**) with the ATP-binding site of Aurora-A kinase (PDB ID: 2W1C).

To obtain a better understanding of the differences in Aurora-A kinase inhibition by JNJ-7706621 versus its sulfonamide analogues, the compounds were docked in silico to the enzyme active site. All the compounds showed close affinity scores compared to JNJ-7706621, < −8.0 kcal/mol. Commensurate with JNJ-7706621, **1b** interacted with key amino acids of ATP binding site, Val-147, Lys-141, Glu 260, Leu 263, and Leu-139). Nevertheless, **1b** showed only one H-bond, with Ala-213, in contrast to JNJ-7706621 which displayed two H-bonds with Ala-213 and with Glu-211, both of which were reported as essential for optimal anti-Aurora-A kinase activity [12], Figure 3A,B. Moreover, the bulky trifluoromethyl groups of **1d** changed the orientation of the external acidic sulfonamide away from the hinge region, affecting its interaction with Ala-213 and with Glu-211, Figure 3C.

## 3. Materials and Methods

General method for the synthesis of compounds (**1a–d**):

The corresponding benzenesulfonyl chloride (3.75 mmol) was added dropwise to a suspension of 4-((3-amino-1*H*-1,2,4-triazol-5-yl)amino)benzenesulfonamide (**5**) (3.75 mmol) in anhydrous pyridine (3 mL). The reaction was stirred at RT overnight. The compounds were then purified either by flash chromatography or by recrystallization from a suitable solvent system.

4-((5-amino-1-(phenylsulfonyl)-1*H*-1,2,4-triazol-3-yl)amino)benzenesulfonamide (**1a**): column chromatography, Silica Gel, Hexane-EtOAc (30:70). Yield: 34%. white solid (MP: 229–231 °C). ^1^H NMR (300 MHz, DMSO-d6): 9.70 (s, 1H), 7.99 (d, *J* = 8.3 Hz, 2H), 7.78 (s, 1H), 7.69 (d, *J* = 8.6 Hz, 4H), 7.60–7.46 (m, 3H), 7.15 (s, 2H). ^13^C NMR (75 MHz, DMSO-d6): 159.77, 157.88, 143.97, 136.32, 135.68, 135.44, 130.19, 127.88, 127.24, 116.39. LC/MS (ESI+) m/z [M + 1] calculated: 395.06, found: 395.44. Elemental analysis, calculated (%): C, 42.63; H, 3.58; N, 21.31; S, 16.26, found (%): C, 43.01; H, 3.56; N, 21.12; S, 15.98.

4-((5-amino-1-((2,6-difluorophenyl)sulfonyl)-1*H*-1,2,4-triazol-3-yl)amino)benzenesulfonamide (**1b**): column chromatography, Silica Gel, Hexane-EtOAc (30:70). Yield: 18.6%. White solid (MP: 224–226 °C). ^1^H NMR (300 MHz, DMSO-d6): 10.97 (s, 1H), 7.87 (d, *J* = 8.3 Hz, 2H), 7.73 (d, *J* = 8.3 Hz, 2H), 7.58 (d, *J* = 8.3 Hz, 2H), 7.52–7.39 (m, 1H), 7.08 (s, 2H), 5.63 (s, 2H). ^13^ C NMR (75 MHz, DMSO-*d*_6_): 159.87, 159.46 (d, *J* = 255.8 Hz), 157.75, 157.47, 143.92, 138.78, 135.81, 127.14, 116.36, 114.50, 114.20. LC/MS (ESI+) m/z [M + 1] calculated: 431.04, found: 431.46. Elemental analysis, calculated (%): C, 39.07; H, 2.81; F, 8.83; N, 19.53; S, 14.90, found (%): C,39.42; H, 2.76; N, 19.13; S, 14.51; F, 8.54.

4-((5-amino-1-tosyl-1*H*-1,2,4-triazol-3-yl)amino)benzenesulfonamide (**1c**): column chromatography, Silica Gel, CH_2_Cl_2_ (100%). Yield: 3.4%. White solid ^1^H NMR (300 MHz, DMSO-d6): 9.68 (s, 1H), 7.86 (d, *J* = 9.0 Hz, 2H), 7.77 (d, *J* = 8.2 Hz, 2H), 7.56 (m, 6H), 7.13 (s, 2H), 6.10 (s, 2H), 2.36 (s, 3H). ^13^C NMR (75 MHz, DMSO-*d*_6_) δ 159.70, 157.87, 146.35, 143.99, 135.63, 133.39, 130.60, 127.91, 127.23, 116.35, 21.58. LC/MS (ESI+) m/z [M + 1] calculated: 409.08, found: 409.47. Elemental analysis, calculated (%): C, 44.11; H, 3.95; N, 20.58; S, 15.70., found (%): C, 44.17; H, 3.89; N, 20.29; S, 15.55.

4-((5-amino-1-((3,5-bis(trifluoromethyl)phenyl)sulfonyl)-1*H*-1,2,4-triazol-3-yl)amino)benzenesulfonamide (**1d**): recrystallized from acetone. Yield: 36.97%. White solid (MP: 241.6–243 °C). ^1^H NMR (300 MHz, DMSO-d6): 9.78 (s, 1H), 8.59 (d, *J* = 9.2 Hz, 2H), 7.67 (d, J = 8.9 Hz, 2H), 7.53 (d, *J* = 8.9 Hz, 1H), 7.16 (s, 2H). ^13^C NMR (75 MHz, DMSO-d6) δ 160.41, 158.00, 143.67, 138.30, 136.10, 132.36, 131.91, 127.19, 124.55 (q, *J* = 255.8 HZ), 116.5. LC/MS (ESI+) m/z [M + 1] calculated: 530.04, found: 531.51 elemental analysis, calculated (%): C, 36.23; H, 2.28; F, 21.49; N, 15.84; S, 12.09, found (%): C, 36.29; H, 2.14; F, 21.40; N, 15.97; S, 11.97.

Biochemical assay:

JNJ-7706621and compounds **1a–c** were prepared as a 5× stock solution in DMSO. The compounds were then tested in a 10-dose IC_50_ mode, with 3-fold serial dilutions at a starting concentration of 100 μM. The assay kinase buffer was used to dilute the Aurora-A kinase to a final concentration of 1.25 nM. The assay reaction was initiated by the addition of freshly prepared master mix to all wells (reaction, control, and blank) to achieve the following final concentrations: 1.25 nM Aurora-A kinase, 10 µM CSox-peptide substrate, 15 µM ATP, and 1 mM DTT in a final reaction volume of 50 µL per well. The reaction wells were mixed, and the relative fluorescence unit (RFU) data were collected every 3 min for 30 min at 30 °C. The used λ_Ex/Em_ of the chelated Mg^2+^ with the Sox was 360/485 nm.

Molecular docking:

In this study, Aurora-A kinase co-crystallized with 4-fluoro-N-(3-(5-(morpholinomethyl)-1*H*-benzo[d]imidazol-2-yl)-1I-pyrazole-4-yl) benzamide (PDB ID 2W1C) was used. This pyrazole-benzimidazole derivative displayed the main binding interactions with the ATP-pocket required to design a classical Aurora-A kinase inhibitor. The re-docking studies were performed for the crystal structure 2W1C as a validation method. AutoDock tools [13] were used to process the enzyme. PyRx [14] was used to perform the docking. Discovery Studio Visualizer [15] was used to visualize and access the docking results.

## 4. Conclusions

This work reported the development of new triazole derivatives as Aurora-A kinase inhibitors. The new compounds displayed important inhibitory activity, as attested by their IC_50_s in the low to submicromolar range. These new derivatives represent promising inhibitors that can be considered for further investigations.

## Figures and Tables

**Table 1:** Inhibitory activity of the developed compounds against Aurora-A kinase.

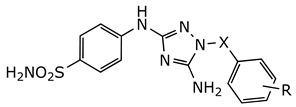			
**Compound**	**X**	**R**	**IC_50_ (µM) ^1^**
JNJ-7706621	CO	2,6-difluoro	0.016 ± 0.00
**1a**	SO_2_	H	0.13 ± 0.06
**1b**	SO_2_	2,6-difluoro	0.21 ± 0.02
**1c**	SO_2_	4-Me	0.23 ± 0.02
**1d**	SO_2_	2,6-ditrifluoromethyl	1.78 ± 0.34

^1^ Data are presented as Mean ± SEM of 3 independent experiments.

## Data Availability

The data presented in this study are available within this article.

## Sample Availability

Not available.
